# Supplementary material for: Electrochemical growth mechanism of nanoporous platinum layers
Source: Commun Chem. 2021 Jun 28;4:98. doi: 10.1038/s42004-021-00535-w (PMC9814644; doi:10.1038/s42004-021-00535-w)
Supplement: Supplementary file 1 — Supplementary Information [file 42004_2021_535_MOESM1_ESM.pdf]

Supplementary Information

Electrochemical Growth Mechanism of Nanoporous Platinum layers

Sarmiza E. Stanca[1*], Oliver Vogt[2], Gabriel Zieger[1], Andreas Ihring[1], Jan Dellith[1], Andreas Undisz[3], Martin Rettenmayr[4], Heidemarie Schmidt[1,5*]

Supplementary Table 1. Pore size and thicknesses of porous platinum layers. Electrolysis of 0.5% $PtCl_4$ in aqueous and non-aqueous media in the absence/presence of additive (AD): $Pb(CH_3COO)_2$ .Resistance: from 4 points contact measurement. Thicknesses results are taken from measurements performed within last 5 years. An unambiguous determination of the thickness of nanoporous layers is only possible for nanoporous layers with constant pore size. The error interval in the thickness increases with increasing differences in bottom porosity, residual porosity, and surface porosity of the nanoporous layer.

Aqueous electrolysis of 0.5%(g/g)$PtCl_4$ at 22°C<br>Cathode(-) :<br>$Pt^{4+} + 4e^- \rightleftarrows Pt^0$; $Pt^{4+} +2e^- \rightleftarrows Pt^{2+}$; $Pt^2 + 2e^- \rightleftarrows Pt^0$<br>$2H^+ + e^- \rightarrow H_2$<br>Anode(+) :<br>$M \rightarrow M^{z+} + ze^-$; $4HO^- \rightarrow O_2 + 2H_2O + 4\ e^-$; $2Cl- \rightarrow Cl_2 + 2e^-$						Non-aqueous electrolysis 0.5% (g/g)$PtCl_4$ at 22°C<br>Cathode(-) :<br>$Pt^{4+} +4\ e^- \rightleftarrows Pt^0$; $Pt^{4+} +2\ e^- \rightleftarrows Pt^{2+}$; $Pt^2 +2\ e^- \rightleftarrows Pt^0$<br>$2H^+ + e^- \rightarrow H_2$<br>Anode(+):<br>$2(CH_3)_2C\text{-}OH \rightarrow 2(CH_3)_2C{=}O + 2H^+ + 2e^-$<br>$M \rightarrow M^{z+} + ze^-$; $4HO^- \rightarrow O_2 + 2H_2O + 4e^-$; $2Cl^- \rightarrow Cl_2 + 2e^-$					
t (s)	AD % g/g	Potential V vs RE	Pore size (nm)	R (kΩ)	Thickness (nm)	T (s)	AD % g/g	Potential V vs RE	Pore size (nm)	R (kΩ)	Thickness (nm)
30	0.00	-0.7	80.5±0.1	30±1	180±10	90	0.00	-0.7	5.1±0.1	821±14	50±2
		-0.8	90.0±0.1	37±2	190±12			-0.8	8.9±0.3	814±10	59±2
		-0.9	90.3±0.1	35±1	200±15			-0.9	9.1±0.3	832±15	63±4
		-1.0	90.5±0.2	32±4	230±15			-1.0	9.3±0.2	829±19	70±5
		-1.1	110.0±0.2	40±4	250±20			-1.1	9.7±0.3	834±18	77±5
30	0.01	-0.7	510.1±2.2	80±1	800±25	90	0.01	-0.7	18.1±0.2	950±31	80±2
		-0.8	550.4±2.0	83±1	820±25			-0.8	20.1±0.3	945±35	85±2
		-0.9	570.5±0.9	79±4	820±25			-0.9	35.2±0.2	948±37	94±7
		-1.0	580.3±1.0	77±3	890±30			-1.0	74.4±0.4	951±39	97±7
		-1.1	585.5±1.2	91±2	900±35			-1.1	80.1±0.3	957±42	97±9
30	0.03	-0.7	545.3±1.5	121±1	980±20	90	0.03	-0.7	18.1±0.1	1045±52	114±2
		-0.8	564.5±1.7	125±3	985±32			-0.8	38.7±0.2	1051±56	120±5
		-0.9	587.1±1.2	128±3	991±34			-0.9	59.1±0.3	1062±61	123±7
		-1.0	589.4±1.8	134±4	993±35			-1.0	78.3±0.2	1071±60	125±7
		-1.1	610.2±2.1	129±4	998±37			-1.1	110.7±0.3	1083±69	127±7
30	0.05	-0.7	592.1±2.9	134±3	990±34	90	0.05	-0.7	40.4±0.1	1290±70	120±3
		-0.8	598.4±1.8	139±3	995±42			-0.8	59.7±0.3	1327±75	124±5
		-0.9	607.2±2.0	141±2	997±44			-0.9	77.7±0.3	1402±78	125±5
		-1.0	630.2±2.7	145±4	999±48			-1.0	118.3±0.5	1450±80	127±5
		-1.1	645.5±2.4	150±4	1005±50			-1.1	120.9±0.7	1520±79	129±7
60	0.01	-0.7	722.1±1.5	154±3	991±32	120	0.01	-0.7	140.4±0.7	1490±68	250±8
		-0.8	724.4±2.4	158±3	995±37			-0.8	159.7±0.7	1521±70	259±10
		-0.9	750.2±2.8	161±2	999±44			-0.9	172.7±0.8	1507±77	265±14
		-1.0	780.2±3.3	165±4	1023±50			-1.0	188.1±0.9	1650±81	274±15
		-1.1	789.5±3.4	168±4	1050±52			-1.1	190.9±0.9	1690±87	287±15
90	0.01	-0.7	802.1±2.5	184±2	1120±40	150	0.01	-0.7	170.4±0.4	1690±60	264±10
		-0.8	814.4±2.8	187±3	1142±45			-0.8	188.7±0.6	1701±67	266±12
		-0.9	843.2±2.9	190±3	1150±47			-0.9	192.7±0.6	1777±69	289±20
		-1.0	850.2±5.3	194±4	1190±50			-1.0	198.1±0.7	1850±71	325±25
		-1.1	889.5±5.4	197±4	1200±51			-1.1	199.5±0.9	1900±82	323±27
120	0.01	-0.7	823.2±3.5	194±3	1260±40	180	0.01	-0.7	190.1±0.5	1890±70	550±19
		-0.8	845.4±3.7	197±3	1285±42			-0.8	198.7±0.6	1899±71	559±21
		-0.9	863.1±4.2	201±4	1291±44			-0.9	202.3±0.8	1905±76	561±30
		-1.0	901.4±6.3	214±7	1293±45			-1.0	207.4±0.9	1956±79	570±35
		-1.1	949.5±7.3	240±7	1298±47			-1.1	229.2±0.9	1994±82	587±37
